# Successful salvage therapy for refractory primary cutaneous gamma-delta T-cell lymphoma with a combination of brentuximab vedotin and gemcitabine

**DOI:** 10.1186/s40164-021-00225-2

**Published:** 2021-05-13

**Authors:** Sophie Voruz, Laurence de Leval, Anne Cairoli

**Affiliations:** 1 Service and Central Laboratory of Hematology, University Hospital Lausanne (CHUV), Rue du Bugnon 46, CH – 1011 Lausanne, Switzerland; 2 Institute of Pathology, Department of Laboratory Medicine and Pathology, Lausanne University Hospital and Lausanne University, Lausanne, Switzerland

**Keywords:** Primary cutaneous gamma-delta T-cell lymphoma, Targeted treatment, Cutaneous T-cell lymphoma, Refractory disease

## Abstract

Primary cutaneous gamma-delta T-cell lymphoma (PCGD-TCL) is a very rare lymphoma with an aggressive clinical course and a dismal outcome. The prognosis is linked to a pronounced resistance to chemotherapy and radiotherapy. No standard treatment approach is defined due to the low frequency of the disease and lack of prospective studies. CD30 is expressed in almost half of the cases of PCGD-TCL, which offers a potential therapeutic option. We report the successful treatment of a 68-year-old man who suffered PCGD-TCL with a combination of Brentuximab Vedotin and Gemcitabine after the failure of two lines of previous chemotherapy. CD30 expression was only partial. The treatment was very well tolerated and allowed the patient to benefit from allogeneic hematopoietic stem cell transplantation.

**Supplementary Information:**

The online version contains supplementary material available at 10.1186/s40164-021-00225-2.

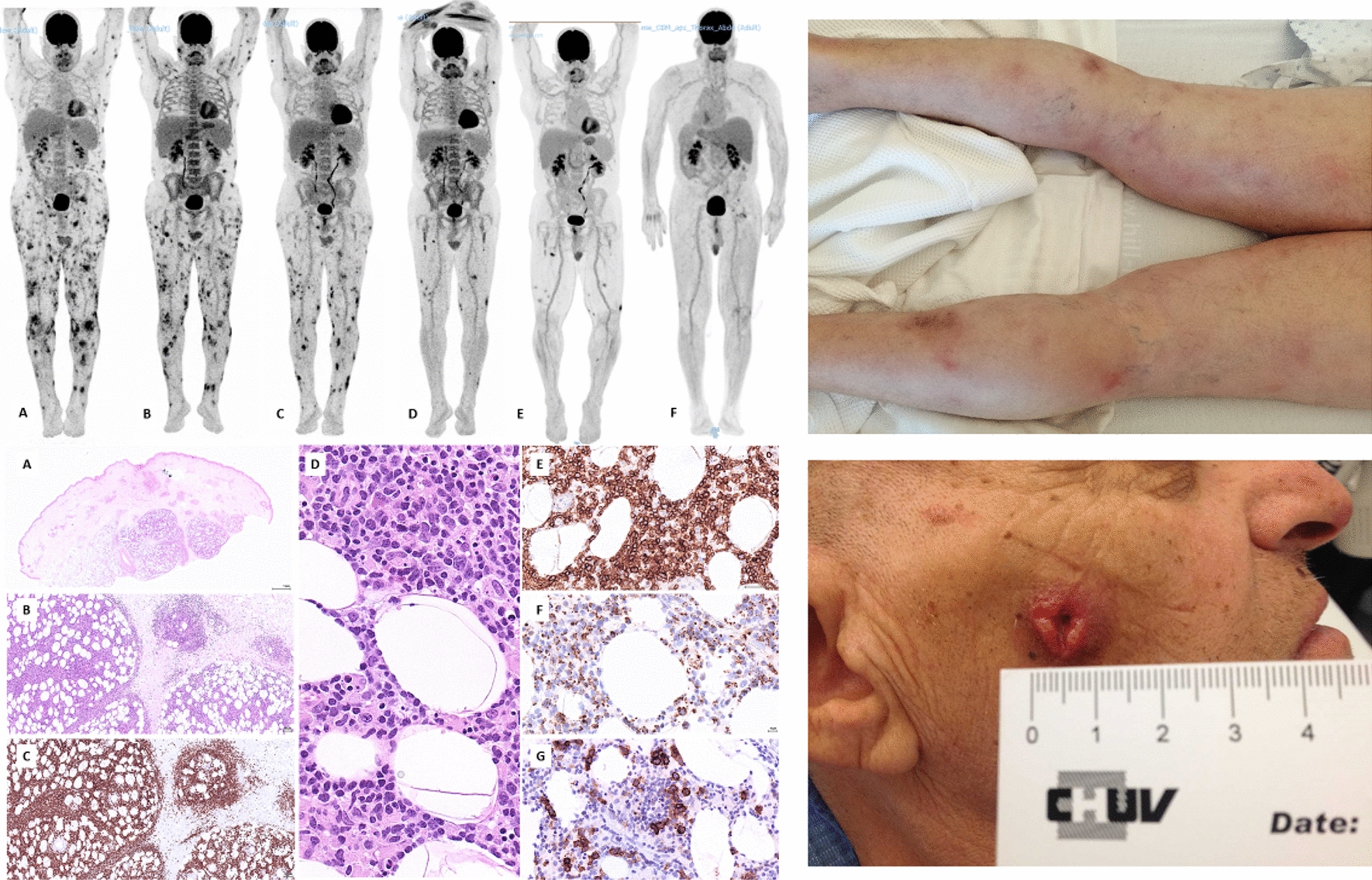

## To the Editor

Primary cutaneous gamma delta T-cell lymphoma (PCGD-TCL) represents < 1% of cutaneous T-cell lymphomas [1]. The clinical course is mostly aggressive with a median survival rate between 11 and 20% at 5 years with ranges from 15 to 31 months after diagnosis [2–4]. The prognosis is even worse for patients with predominantly subcutaneous fat involvement, as in this case, compared to those with epidermal or dermal disease only, reaching 10 months [2]. The prognosis is linked to a pronounced resistance to chemotherapy and radiotherapy. No standard treatment approach is defined due to the low frequency of the disease and lack of prospective studies. Here, we report the case of a patient with refractory PCGD-TCL who was successfully treated with a combination of Brentuximab Vedotin and Gemcitabine.

**Fig. 1:**
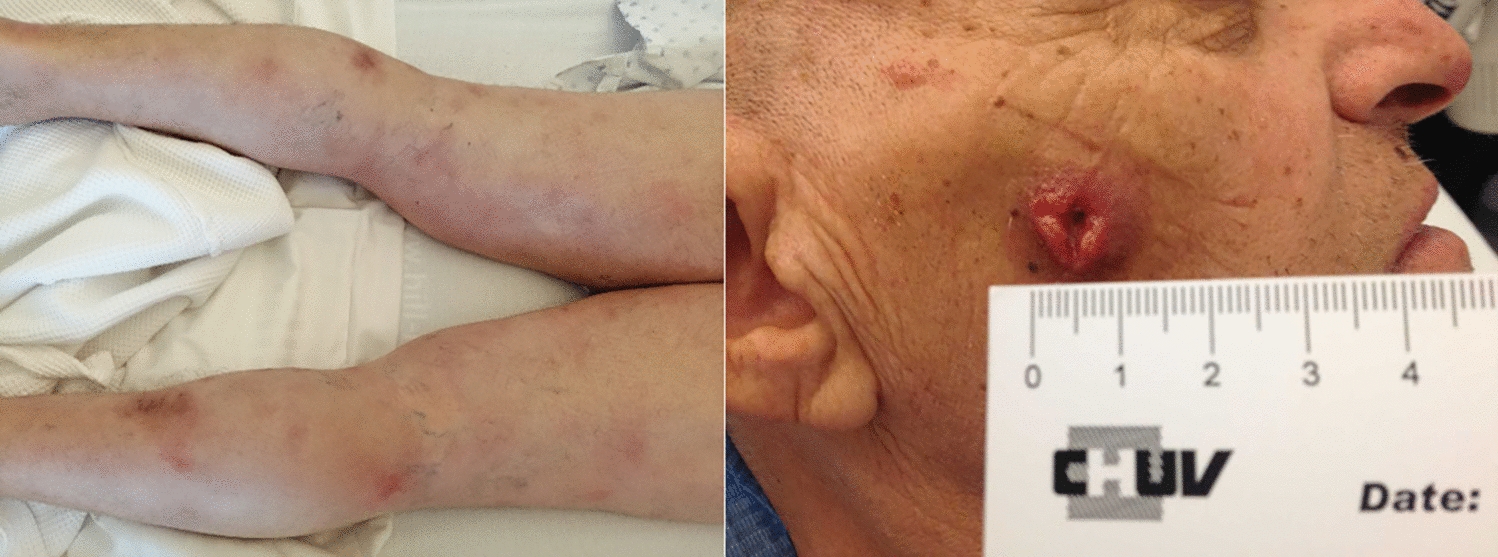
The panniculitis-like lesions with adjacent erythematous patches and skin ulceration

A 68-year-old man presented with numerous panniculitis-like thickened erythematous red-brownish painless cutaneous plaques that had developed over the past 3 months. Some lesions had evolved with skin ulceration. They were first noticed on the lower extremities and subsequently involved the trunk, the upper extremities and the face (Fig. 1). The patient reported no other symptoms and particularly no pruritus. PET scan showed multiple subcutaneous metabolic lesions with maximum standard uptake values (SUV) of 12.3 mg/l, without hepatosplenic or systemic involvement.

**Fig. 2:**
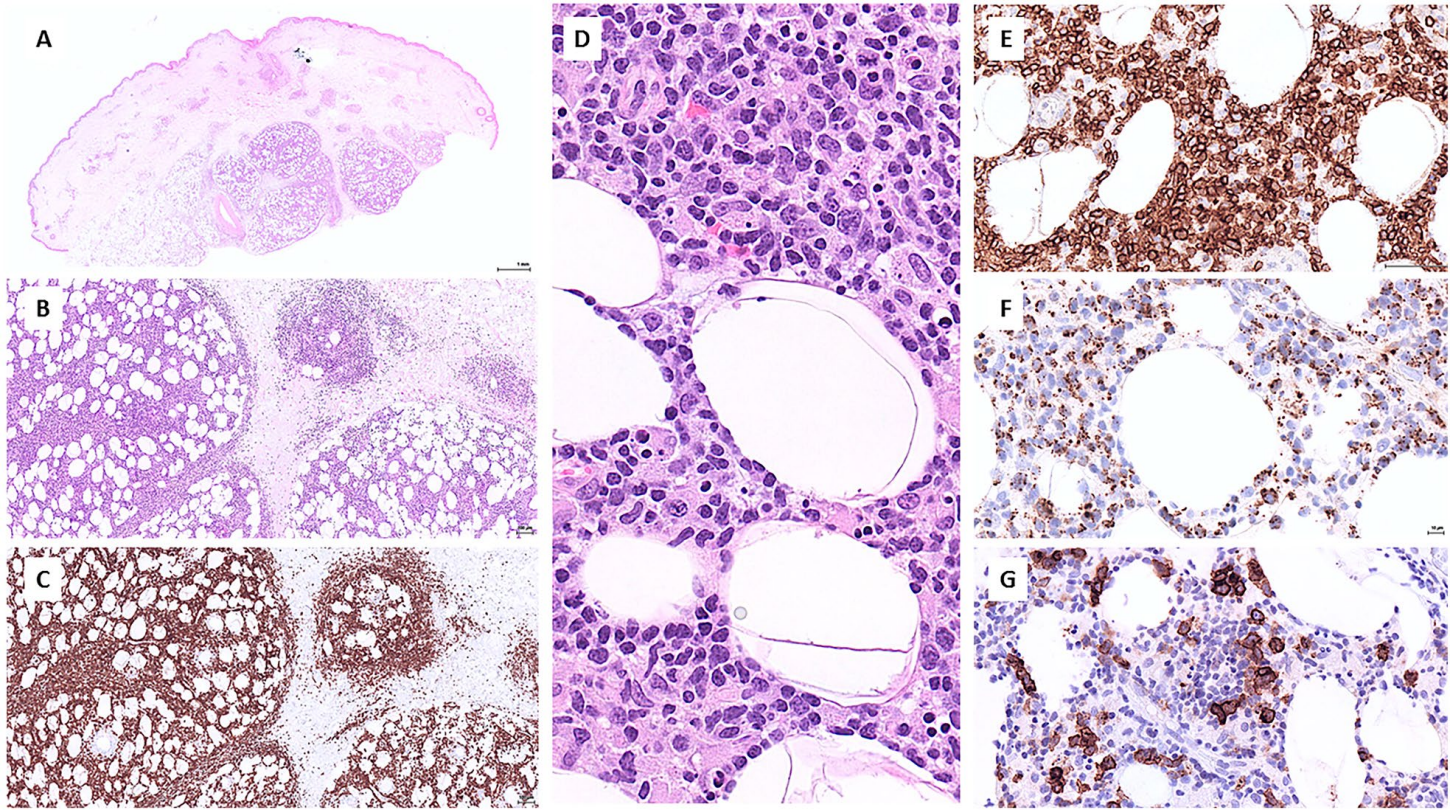
Histopathology of a skin biopsy. **A** Panoramic view showing an infiltrate predominantly involving the subcutaneous fat lobules. **B** Subcutaneous infiltrate with a panniculitis-like pattern, and focal dermal involvement. **C** CD3 immunostaining showing diffuse CD3 positivity and highlighting the subcutaneous and dermal distribution of the infiltrate. **D** High-power view of the subcutaneous fat showing an infiltrate of medium to large atypical lymphoid rimming the adipocytes and admixed to histiocytes. **E** Strong expression of TCR delta by the T cells. **F** Strong expression of perforin by the T cells. **G** Partial expression of CD30 by the T cells

A biopsy of a calf lesion showed a lymphoid infiltrate involving the subcutaneous fat and extending to the dermis while preserving the epidermis. The infiltrate consisted of medium to large atypical lymphocytes admixed with a large number of histiocytes, featuring fat cell rimming and producing a panniculitis-like pattern (Fig. 2). By immunohistochemistry the lymphoid cells were positive for CD2 and CD3, weakly positive for CD56, and expressed several cytotoxic markers (TIA-1 granzyme B, and perforin). They were positive for TCR delta and negative for TCR betaF1, CD4, CD8, CD5, CD7 and CD57. Moderate to strong CD30 expression was detected in a subset of the lymphoid cells (< 50%). Ki67 proliferation fraction was 80%. PCR studies documented a monoclonal rearrangement of TRG and TRB genes.

A diagnosis of PCGD-TCL was established, and the patient was administered CHOEP chemotherapy and prophylactic intrathecal chemotherapy. After 2 cycles, PET-CT showed progressive disease, and the patient developed B symptoms. Therapy was switched to B-CHP; after two cycles, no improvement was clinically observed and the PET-CT showed only a slight response of some of the lesions (Deauville score 5). The patient condition worsened with ECOG 3. A third-line therapy was initiated with brentuximab-vedotin (1.8 mg/kg D1) and gemcitabine (800 mg/m^2^ D1 and D8). After two cycles, we observed a very good partial remission and the patient received four additional cycles very well tolerated, and recovered an excellent general condition. After 6 cycles, clinical response was almost complete; PET-CT scan showed very few persistent lesions with a SUVmax < 3.5 mg/l in the upper limbs and trunk that were difficult to interprete as residual tumor versus inflammatory response. Nine months after the diagnosis, an allogeneic stem cell transplantation (ASCT) with a haplo-identical donor was performed after sequential clofarabine-based conditioning regimen. The patient is still in clinical complete response 9 months after the transplant.

Anthracycline-based treatment is often use to treat PCGD-TCL despite disappointing results [5]. ASCT has shown favorable and durable outcome for some patients [5–9], however maximal lowering of the disease burden is still necessary before proceeding to transplant. As CD30 is expressed in almost half of the cases of PCGD-TCL [10], brentuximab-vedotin is a potential therapeutic option. Recently, brentuximab-vedotin has shown efficacy in four patients, even if the expression of CD30 was low [11]. Of note, CD30 expression was partial in our case. Weekly gemcitabine is an old drug regimen, very well tolerated in monotherapy, that has been used in a relapsed or refractory setting in other cutaneous T-cell lymphomas with notable efficacy. We thought to use the combination of these two drugs as it was successfully done in pediatric refractory Hodgkin lymphoma [12], since we needed a well-tolerated regimen at this point of the treatment.

The advantage of this treatment was its high tolerability and its efficacy allowing the patient to benefit from ASCT.

Further clinical studies are needed to evaluate better the efficiency of this treatment and its ability to bridge to an ASCT in this rare T-cell lymphoma entity.

- CHOEP regimen: cyclophosphamide, 750 mg/m2 i.v.; doxorubicin, 50 mg/m2 i.v., vincristine, 2 mg i.v on day 1; etoposide, 100 mg/m2/d i.v. on day 1 to 3 and prednisone, 100 mg/d p.o. on day 1 through 5 every 21 days.
- Prophylactic intrathecal chemotherapy: methotrexate 15 mg, cytarabine 30 mg, methylprednisolone 30 mg.
- B-CHP regimen: brentuximab-vedotin, 1.8 mg/kg i.v.; cyclophosphamide, 750 mg/m2 i.v. and doxorubicin, 50 mg/m2 i.v. on day 1; prednisone 100 mg/d p.o. on day 1 to 5, every 21 days.

## Supplementary Information

**Additional file 1: Figure S1.** PET-CT scan, ^18^F-FDG. **A** At diagnosis. **B** After 2 cycles of CHOEP. **C** After 2 cycles of B-CHP. **D** After 2 cycles of B-gemcitabine. **E** After 6 cycles of B-gemcitabine (pre-transplant). **F** Nine months after allotranplant.

## Data Availability

Not applicable.
