# Supplementary figures and images for: Successful salvage therapy for refractory primary cutaneous gamma-delta T-cell lymphoma with a combination of brentuximab vedotin and gemcitabine
Source: Exp Hematol Oncol. 2021 May 13;10:32. doi: 10.1186/s40164-021-00225-2 (PMC8117509; doi:10.1186/s40164-021-00225-2)

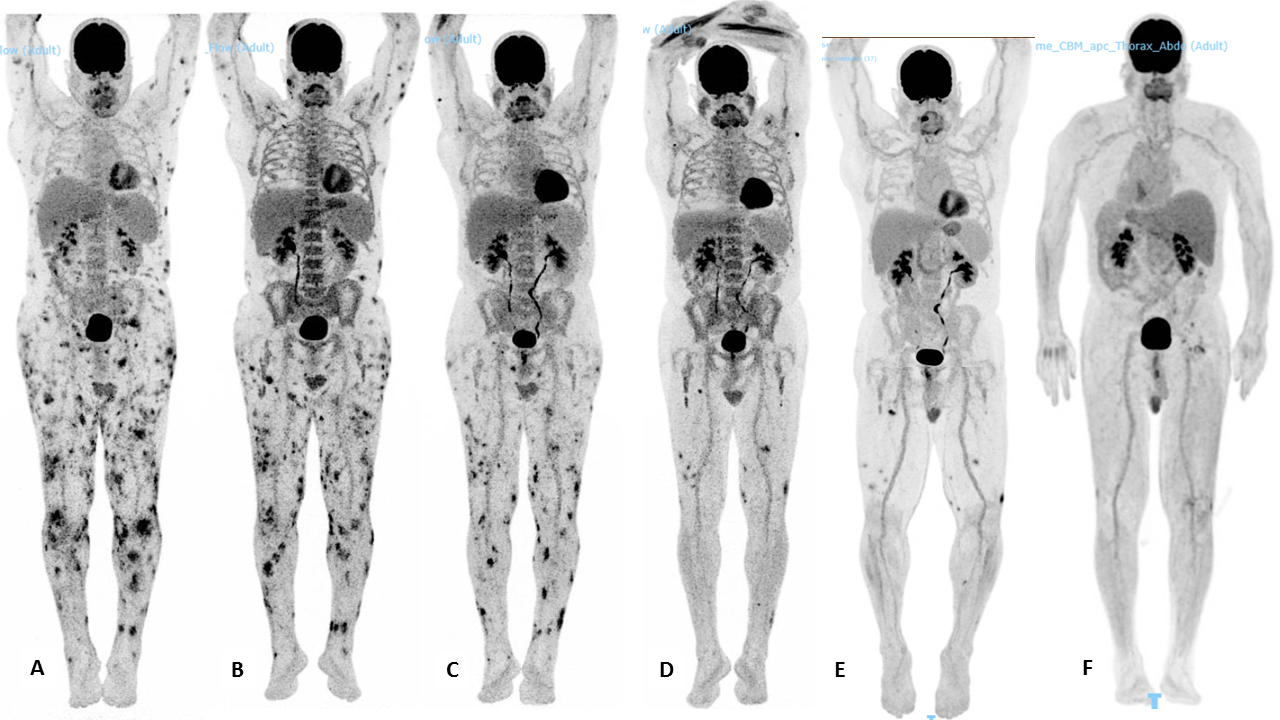

Supplement: Supplementary file 1 — Additional file 1: Figure S1. PET-CT scan, 18F-FDG. A At diagnosis. B After 2 cycles of CHOEP. C After 2 cycles of B-CHP. D After 2 cycles of B-gemcitabine. E After 6 cycles of B-gemcitabine (pre-transplant). F Nine months after allotranplant. [file 40164_2021_225_MOESM1_ESM.tif]
